# MiR-26a-5p as a useful therapeutic target for upper tract urothelial carcinoma by regulating WNT5A/β-catenin signaling

**DOI:** 10.1038/s41598-022-08091-6

**Published:** 2022-04-28

**Authors:** Yueh-Hua Chung, Yuan-Tso Cheng, Ying-Hsien Kao, Wan-Chi Tsai, Gong-Kai Huang, Yen-Ta Chen, Yuan-Chi Shen, Ming-Hong Tai, Po-Hui Chiang

**Affiliations:** 1 Department of Urology, Kaohsiung Chang Gung Memorial Hospital and Chang Gung University College of Medicine, Kaohsiung, 83301 Taiwan, ROC; 2 Department of Medical Laboratory Science and Biotechnology, Kaohsiung Medical University, Kaohsiung, Taiwan, ROC; 3 Institute of Biomedical Sciences, National Sun Yat-Sen University, Kaohsiung, 80424 Taiwan, ROC; 4 Department of Medical Research, E-Da Hospital, Kaohsiung, 82445 Taiwan, ROC; 5 Department of Pathology, Kaohsiung Chang Gung Memorial Hospital and Chang Gung University College of Medicine, Kaohsiung, 83301 Taiwan, ROC

**Keywords:** Cancer, Molecular biology

## Abstract

The role of miRNAs in cancer and their possible function as therapeutic agents are interesting and needed further investigation. The *miR-26a-5p* had been demonstrated as a tumor suppressor in various cancers. However, the importance of *miR-26a-5p* regulation in upper tract urothelial carcinoma (UTUC) remains unclear. Here, we aimed to explore the *miR-26a-5p* expression in UTUC tissues and to identify its regulatory targets and signal network involved in UTUC tumorigenesis. The *miR-26a-5p* expression was validated by quantitative real-time polymerase chain reaction (qPCR) using renal pelvis tissue samples from 22 patients who were diagnosed with UTUC and 64 cases of renal pelvis tissue microarray using in situ hybridization staining. BFTC-909 UTUC cells were used to examine the effects of *miR-26a-5p* genetic delivery on proliferation, migration and expression of epithelial-to-mesenchymal transition (EMT) markers. *MiR-26a-5p* was significantly down-regulated in UTUC tumors compared to adjacent normal tissue and was decreased with histological grades. Moreover, restoration of *miR-26a-5p* showed inhibition effects on proliferation and migration of BFTC-909 cells. In addition, *miR-26a-5p* delivery regulated the EMT marker expression and inhibited WNT5A/β-catenin signaling and expression of downstream molecules including NF-κB and MMP-9 in BFTC-909 cells. This study demonstrated that *miR-26a-5p* restoration may reverse EMT process and regulate WNT5A/β-catenin signaling in UTUC cells. Further studies warranted to explore the potential roles in biomarkers for diagnostics and prognosis, as well as novel therapeutics targets for UTUC treatment.

## Introduction

Urothelial carcinoma (UC) is a common cancer of the epithelium lining the urinary tract, among upper urinary tract cancers, including renal pelvis carcinoma and ureteral carcinoma as known as upper tract urothelial carcinoma (UTUC). Despite of a relatively uncommon malignancy in Western countries, UTUC represent more than 40% of UCs in Taiwan^1^. In addition, advanced UTUC is often associated with poor oncologic outcomes^2^. Therefore, UTUC is indeed a crucial health problem in Taiwan and developing novel therapeutic and diagnostic strategies for the treatment is necessary.

MicroRNAs (miRNAs) are a class of small non-coding RNAs (19–22 nucleotides in length) that regulate gene expression at a post-transcriptional level via complementarily binding to their mRNA targets^3^. It is well known that miRNAs are involved in diverse biological processes, including cell differentiation, proliferation, and apoptosis^3^. Numerous miRNAs have been reported to display tumor suppressor activity in various cancers and may act as a potential target for treatment, including *miR-26a*^4–9^. There was a prostate cancer study showing that miR-26a was markedly reduced in cancer tissues and acted in concert to regulate genes that promote metastasis^10^. Moreover, studies had showed that miR-26a overexpression inhibits the tumor cell growth both in vitro and in vivo^11^. These findings support that miR-26a is a potential target of cancer therapy.

Epithelial-to-mesenchymal transition (EMT) is a process for the transformation of cancer cells from epithelial to mesenchymal types, which is clinically correlated with cancer metastasis and associated with recurrence and drug resistance in cancers^3,12–18^. Thus, suppressing EMT process could inhibit the migration and invasion during cancer progression. Additionally, there was study showing that reduced miR-26a expression is associated with lung metastasis and poor overall survival of osteosarcoma patients^11^.

To date, whether miR-26a (*miR-26a-5p*) regulates EMT process of UTUC cells and the molecular mechanisms involved remain unclear and need further investigation. The aims of this study were first to validate the *miR-26a-5p* expression using UTUC samples. Subsequently, we investigated the tumor suppressor function and mechanism using a renal pelvis transitional cell carcinoma cell line (BFTC-909 cells)^3^.

## Results

### miR-26a-5p expression was down-regulated in human UTUC tissues

**Figure 1:**
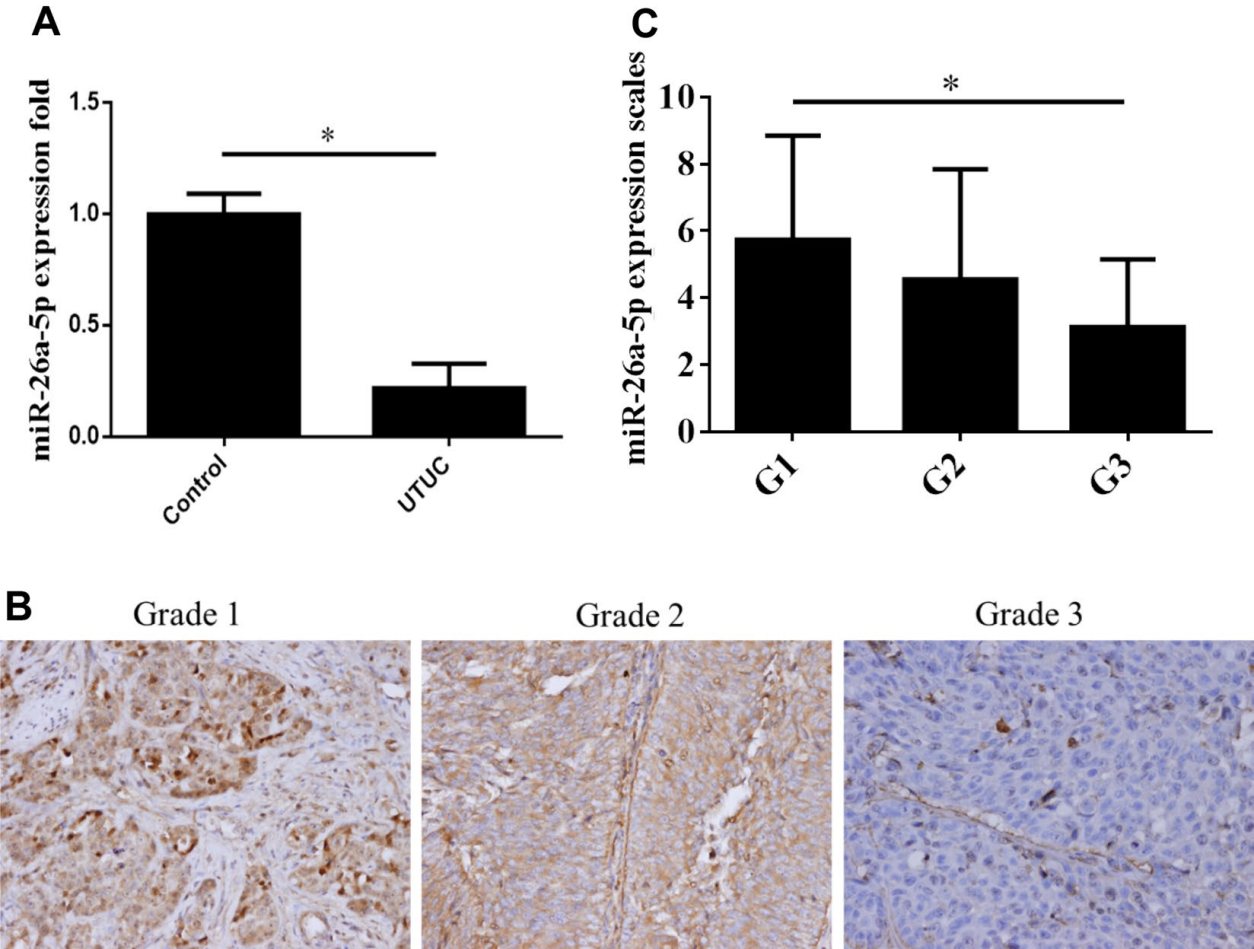
Expression profiles of *miR-26a-5p* in human UTUC samples. (**A**) Down-regulation of *miR-26a-5p* in UTUC tumor tissues (n = 22) compared with adjacent normal control (n = 14) by RT-qPCR analysis. (**B**) In situ hybridization (ISH) analysis of *miR-26a-5p* expression in TMA of human renal pelvis carcinoma, including Grade 1 (G1; n = 23), Grade 2 (G2; n = 33) and Grade 3 (G3; n = 8) samples. (**C**) Quantification of *miR-26a-5p* expression levels from ISH staining results. Data represent the mean ± SEM. *P < 0.05 compared between the indicated groups.

According to our previous study^3^, several microRNAs were down-regulated in UTUC tissues compared with adjusted normal controls, including *miR-26a-5p*. In this study, we first validated the *miR-26a-5p* expression in UTUC samples using qPCR assay and tissue microarray (TMA) using in situ hybridization staining (ISH). As shown in Fig. 1A, *miR-26a-5p* was significantly down-regulated in UTUC samples (n = 22:14). Thereafter, we next assessed *miR-26a-5p* expression in renal pelvis transitional cell carcinoma TMA which contained 64 tissue sections. As shown in Fig. 1B, *miR-26a-5p* was expressed around cytoplasmic area and the ISH immunosignals decreased progressively in advanced tumor grades. The intensity score was significantly lower in grade 3 tumors than grade 1 tumors (Fig. 1C). Taken together, these data imply the tumor suppressor role of *miR-26a-5p* in UTUC tumorigenesis.

### Restoration of miR-26a-5p reduced BFTC-909 cells proliferation and migration

**Figure 2:**
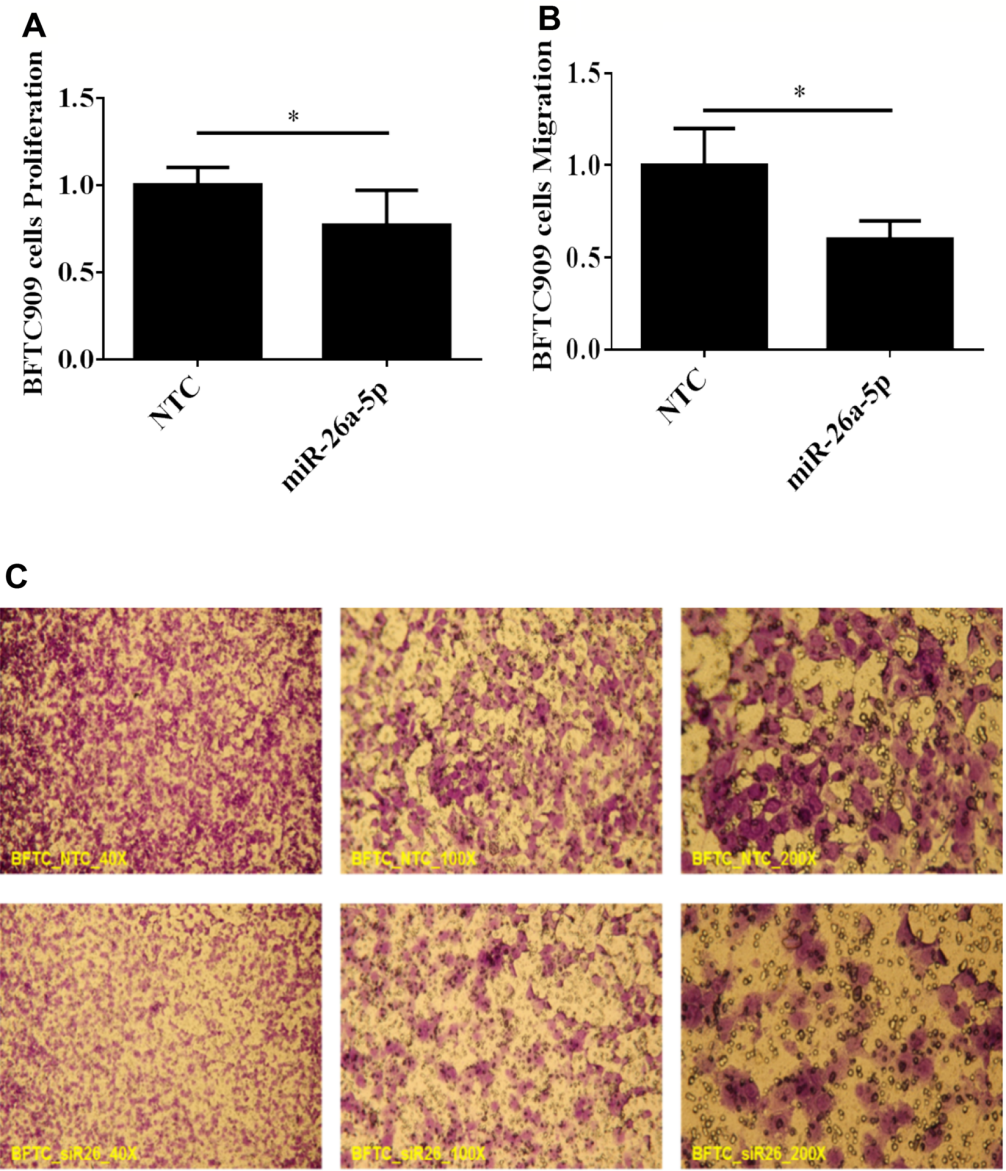
Restoration of *miR-26a-5p* suppressed proliferation and migration of UTUC cells. BFTC-909 UTUC cells were transfected with *miR-26a-5p* mimics, followed by WST-1 and trans-well assays. NTC denotes non-transfection controls with scrambled mimetic treatment. (**A**) After mimetic transfection for 48 h, WST-1 assay was used to evaluate cell numbers. WST-1 value is directly proportional to cell number. (**B**) After mimetic transfection for 72 h, trans-well migration assay was performed to evaluate cell migration ability. (**C**) The crystal violet-stained cells are those penetrating the trans-well membrane. All data are shown as mean ± SEM from three independent experiments. *Indicates a *P* < 0.05 between the indicated groups.

According to the ISH data, we further investigate the effects of *miR-26a-5p* restoration on UTUC cell proliferation and migration. Thus, we transfected *miR-26a-5p* mimics into BFTC-909 cells which is a well-known in vitro model of UTUC^2,3^. The proliferation was exam by Water-soluble tetrazolium (WST-1) assay and migration ability was exam by trans-well assay, respectively. As showed in Fig. 2, the *miR-26a-5p* restoration significantly reduced cell number (Fig. 2A) and migration ability (Fig. 2B,C). According to these results, we demonstrated that *miR-26a-5p* could serve as a tumor repressor in UTUC cell growth and migration.

### Restoration of miR-26a-5p inhibited EMT processes

**Figure 3:**
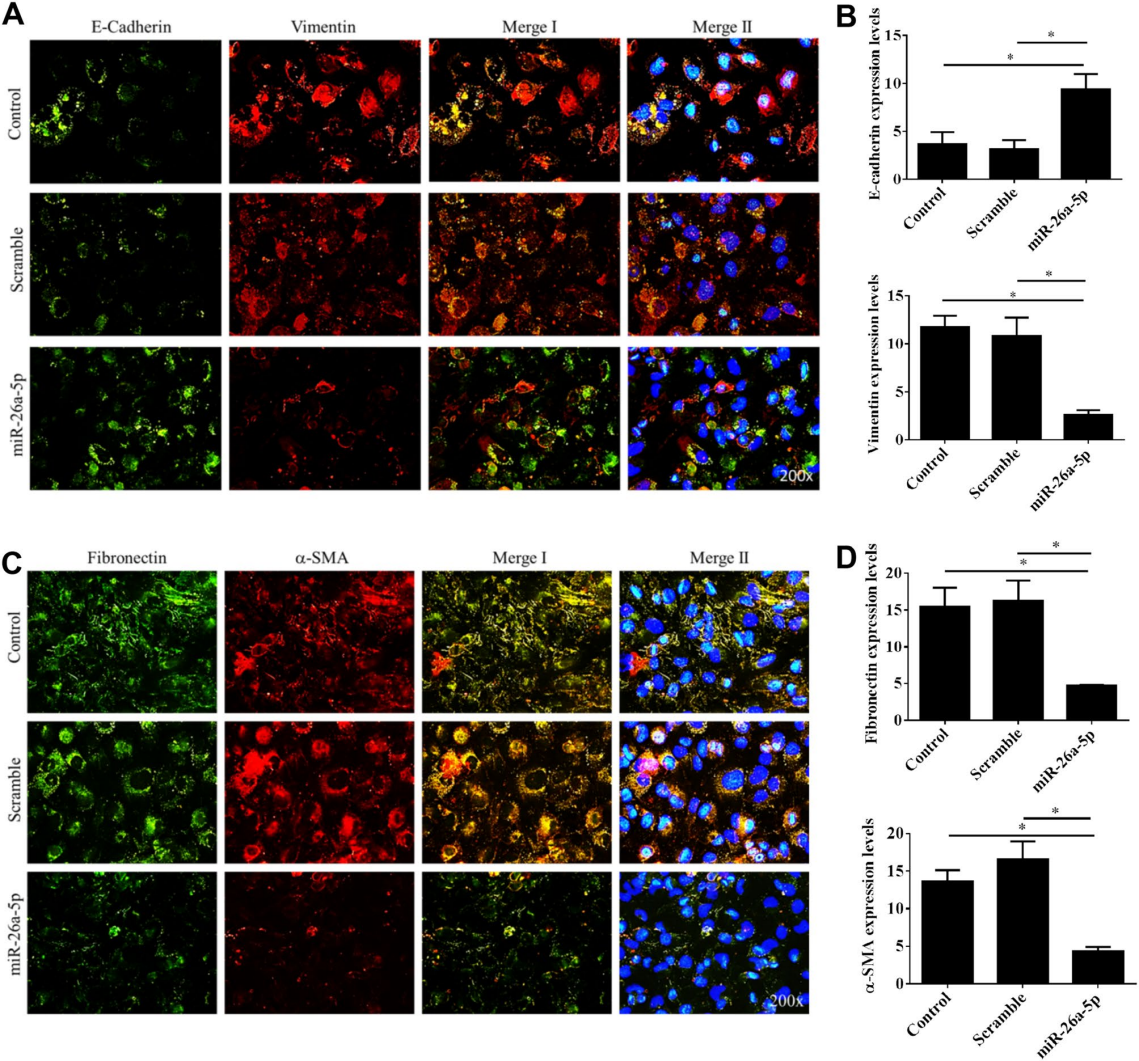
*miR-26a-5p* overexpression inhibited expression of mesenchymal markers in UTUC cells. BFTC-909 UTUC cells were transfected with *miR-26a-5p* mimics for 48 h and subjected to immunofluorescent staining. (**A**) Representative images of cellular E-cadherin (green), vimentin (red), and nuclei (blue) were shown. (**C**) Alternatively, cellular fibronectin (green), α-SMA (red), and nuclei (blue) were immunofluorescently visualized. (**B,D**) The fluorescence intensities of epithelial marker E-cadherin as well as mesenchymal markers, vimentin, α-SMA and fibronectin, were quantified by counting 5–10 different fields per sample. Data are expressed as mean ± SEM (n = 3). *Indicates a *P* < 0.05 between the indicated groups. Original magnification: × 200.

**Figure 4:**
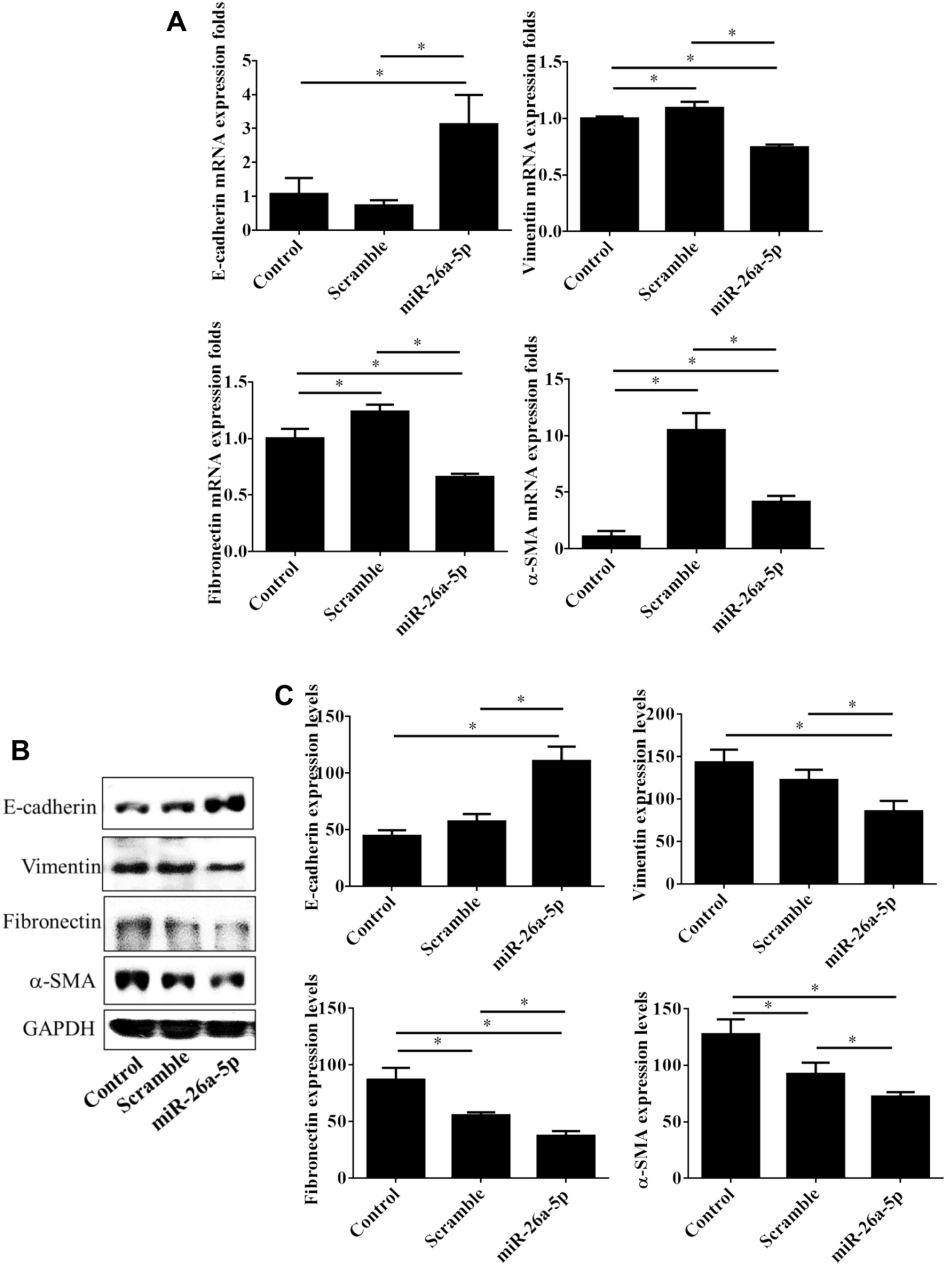
*miR-26a-5p* overexpression inhibited expression of epithelial-to-mesenchymal transition markers in UTUC cells. BFTC-909 UTUC cells were transfected with *miR-26a-5p* mimics for 48 h and subjected to mRNA and protein measurements. Expression levels of epithelial marker E-cadherin and mesenchymal markers including vimentin, fibronectin, and α-SMA were assayed with qPCR (**A**) and western blot (**B**), respectively. GAPDH was used as loading internal control. (**C**) The blotting densitometry of protein levels in BFTC-909 cells. Data are expressed as mean ± SEM (n = 3). *Indicates a *P* < 0.05 between the indicated groups.

Epithelial mesenchymal transition processes has been implicated in carcinogenesis and confers metastatic properties^12^. Thus, we analyzed the effect of *miR-26a-5p* restoration in the EMT processes in UTUC while the E-cadherin, vimentin, α-SMA and fibronectin expression levels were assayed. As showed in Fig. 3, *miR-26a-5p* significantly restored cellular epithelial E-cadherin expression and inhibited α-SMA, fibronectin and vimentin expression. Moreover, the qPCR and western blot analyses similarly showed *miR-26a-5p* restoration significantly enhanced E-cadherin expression in mRNA and protein levels. Conversely, *miR-26a-5p* restoration reduced α-SMA, fibronectin and vimentin expression (Fig. 4). Together, these results indicated that *miR-26a-5p* might prohibit UTUC metastasis by regulating the EMT processes in UTUC cells.

### miR-26a-5p overexpression inactivation of WNT5A/β-catenin signaling in UTUC cells

**Figure 5:**
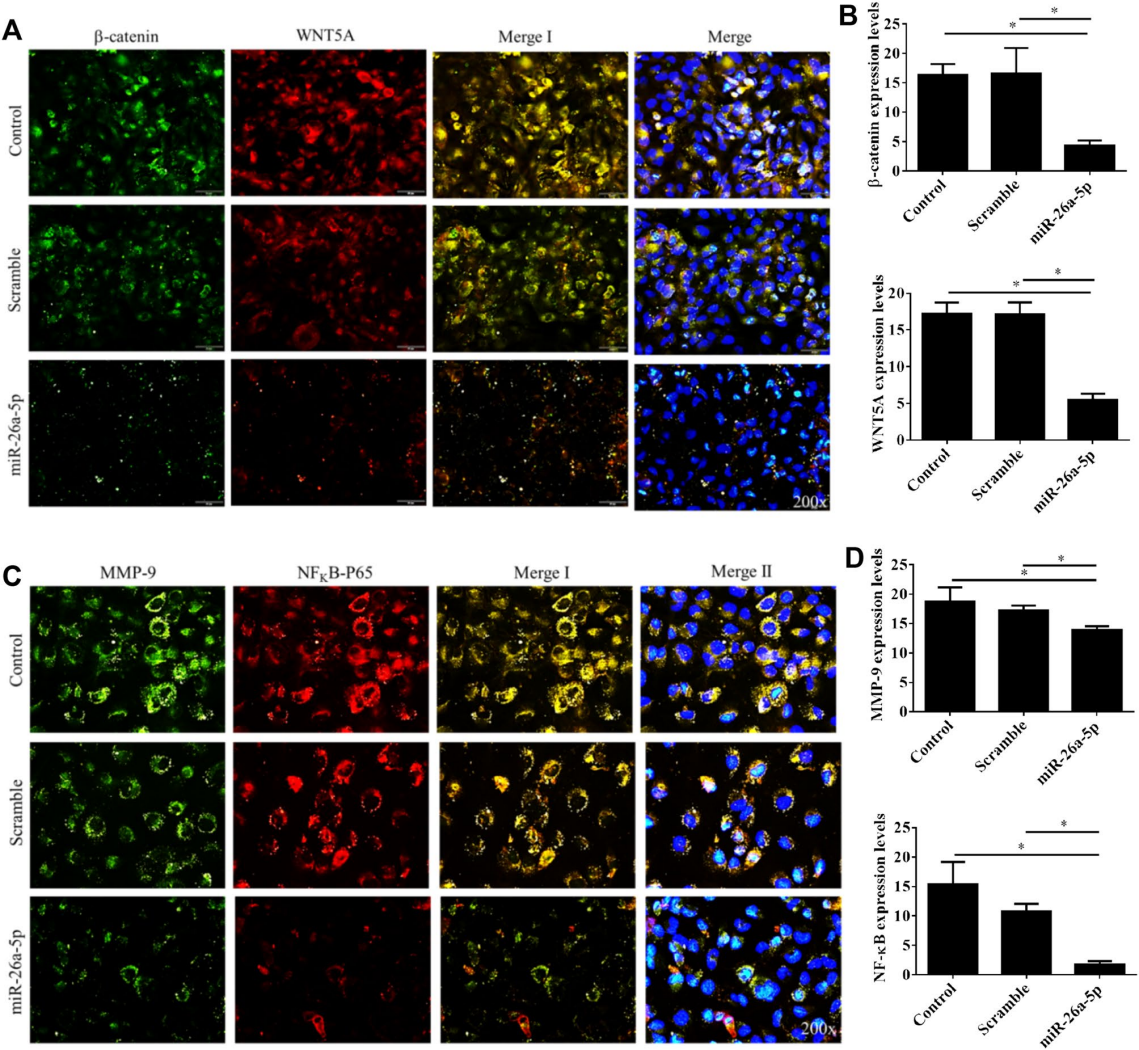
WNT5A/β-catenin signaling and downstream molecules were inhibited by the *miR-26a-5p* overexpression in UTUC cells. BFTC-909 cells were transfected with *miR-26a-5p* mimics for 48 h and subjected to immunofluorescent staining. Representative images for cellular distributions of (**A**) β-catenin (green), WNT5A (red), and nuclei (blue) were shown. (**C**) Alternatively, cellular distributions of MMP-9 (green), NF-κB p65 (red), and nuclei (blue) were immunofluorescently visualized. The fluorescence intensities of β-catenin and WNT5A (**B**), MMP-9 and NF-κB (**D**) were quantified by counting 5–10 different fields per sample. Data are expressed as mean ± SEM (n = 3). *Indicates a *P* < 0.05 between the indicated groups. Original magnification: × 200.

**Figure 6:**
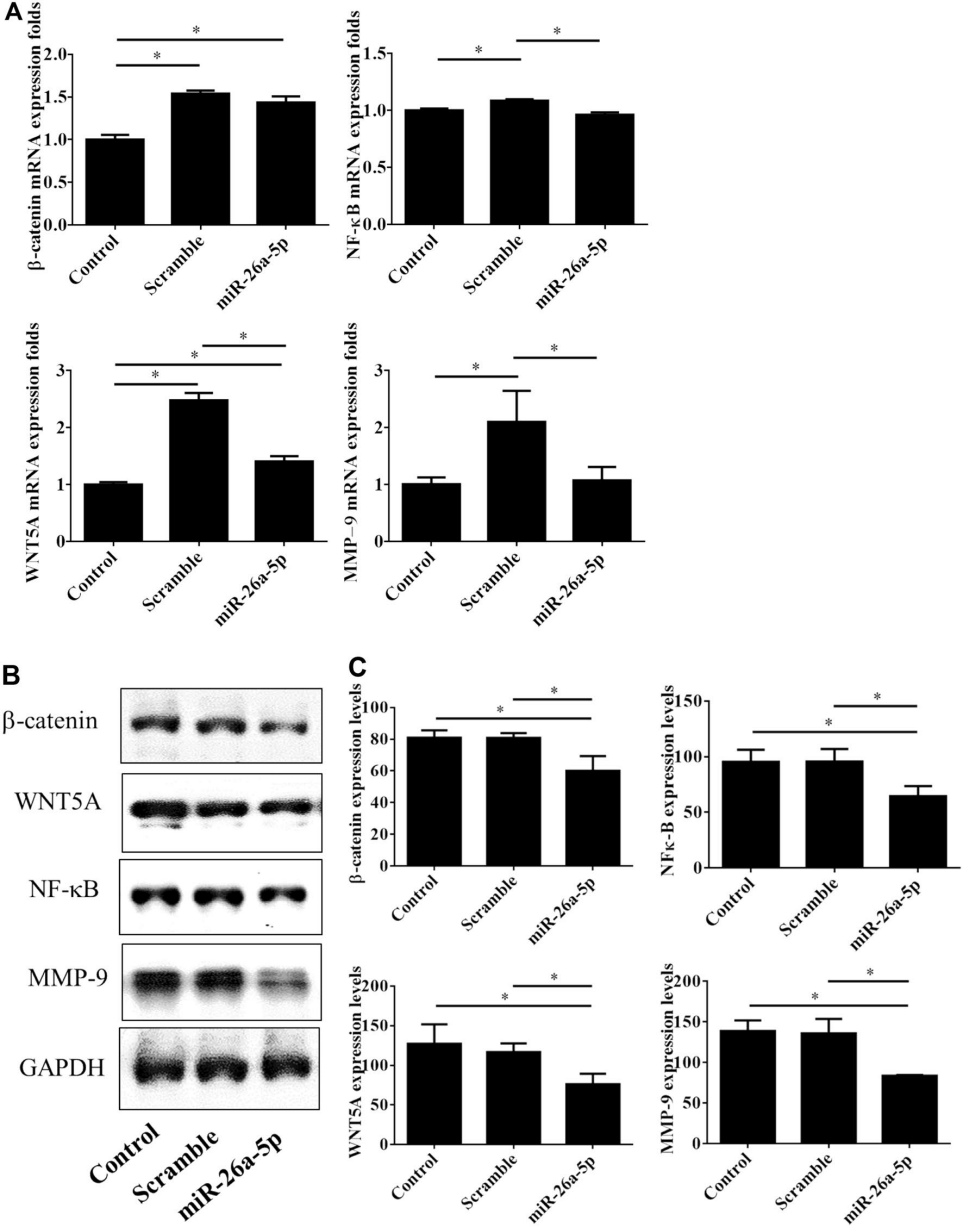
*miR-26a-5p* overexpression modulated expression of Wnt5A/β-catenin signaling mediators and downstream molecules in UTUC cells. BFTC-909 cells were transfected with *miR-26a-5p* mimics for 48 h and subjected to detection of mRNA and protein expression by using qPCR (**A**) and western blot (**B)**, respectively. GAPDH was used as loading internal control. (**C**) The blotting densitometry of protein levels in BFTC-909 cells. Data are expressed as mean ± SEM (n = 3). *Indicates a *P* < 0.05 between the indicated groups.

The oncogenic mechanism underlying *miR-26a-5p* remains unclear. Because WNT5A/β-catenin signaling has been delineated to participate in the signal transduction of *miR-26a-5p*^6,19,20^, we compared the expression profiles of WNT5A/β-catenin signaling mediators between the UTUC cells with and without *miR-26a-5p* gene delivery. By using immunofluorescence staining, the data showed that *miR-26a-5p* restoration down-regulated the β-catenin and WNT5A expression (Fig. 5A,B) in UTUC cells. Furthermore, qPCR and immunoblot analyses confirmed that *miR-26a-5p* restoration reduced the mRNA and protein levels of β-catenin and WNT5A expression in UTUC cells (Fig. 6).

### miR-26a-5p overexpression inhibited NF-κB and MMP-9 expression in BFTC-909 cells

Previous studies have showed that WNT/β-catenin pathway inhibition has the potential to reduce cancer invasion and metastasis via regulating involves NF-κB/MMP-9 mediated EMT process^15,21^. Thus, we further investigated the regulatory role of *miR-26a-5p* in NF-κB (p65) and MMP-9 expression in UTUC cells. Immunofluorescence analysis again demonstrated that *miR-26a-5p* overexpression significantly inhibited the NF-κB and MMP-9 expression in BFTC-909 UTUC cells (Fig. 5C,D). These data were further confirmed by qPCR and western blot analyses (Fig. 6). These results demonstrated that *miR-26a-5p* delivery might block the EMT processes and prevent invasion and metastasis of UTUC cells. In summary, *miR-26a-5p* mechanistically inhibits the EMT process through suppression of WNT5A/β-catenin pathway during UTUC tumorigenesis. Thus, *miR-26a-5p* might be a good therapeutic target for UTUC treatment.

## Discussion

Aberrant miRNA expression have been regarded a hallmark in all cancers, which disrupts the normal function of their targets and leads to the validation of tumor phenotypic transformation and metastasis as well as to drug resistance^22^. Thus, to fine out the gene regulatory effects of miRNAs may help exploit the potential treatment strategy in cancer diseases. There are studies suggesting that suppression of oncogenic miRNAs could become a reliable tool for improving the cancer therapy^3,8,22^.

*miR-26a* has been reported to act as tumor suppressor via targeting specific downstream genes in several human cancers, such as melanoma, thyroid, prostate cancer, laryngeal squamous cell and hepatocellular carcinoma^6,23–29^. However, the role of *miR-26a-5p* in UTUC tumorigenesis was largely unknown. In the present study, we report for the first time that *miR-26a-5p* expression was significantly down-regulated in human UTUC tissues compared with adjacent normal tissues. Moreover, the immunoexpression of *miR-26a-5p* decreased with higher T stage. The intensity score was lower in T3 tumors than T1 and T2 tumors (data not shown). In addition, the similar trends were found when compared to histological grade. This observation supports that high-grade tumor cells often constitutively express lower *miR-26a-5p* than low-grade tumor cells. Given that high histological grade is significantly related to aggressiveness and poor prognosis of tumor, our findings demonstrated that *miR-26a-5p* may play a tumor-suppressive role in the UTUC development. Consistent to our observation, the low *miR-26a-5p* expression has been earlier reported and identified as a poor prognostic marker in colorectal cancer due to poorer overall survival^30^. Therefore, it indicated a potential prognostic and therapeutic value of *miR-26a-5p* in UTUC. This is the first study to assay the *miR-26a-5p* expression in UTUC tissue samples. Moreover, our in vitro study showed that *miR-26a-5p* overexpression significantly suppressed the proliferation, migration and invasion of BFTC-909 cells. Furthermore, *miR-26a-5p* overexpression showed the inhibitory effect on EMT process which is associated with metastasis activity in UTUC cells. These results confirmed the inhibitory effect of *miR-26a-5p* on growth and metastasis in UTUC.

Metastasis is one of the major causes of mortality in UC patients^31^. Therefore, the inhibition of metastasis is an important issue in UC research, including UTUC. There are studies showing that inactivation of NF-κB and the inhibition of the expression of MMP-9, ultimately suppressing invasion and metastasis^31,32^. In addition, it has been reported that high MMP-9 expression levels are associated with clinically aggressive tumors and worse prognosis^33–35^. In the current study, we had provided the evidence of *miR-26a-5p* inhibited the NF-κB and MMP-9 expression. Thus, *miR-26a-5p* might regulate UTUC via suppressing invasion and metastasis.

It is well known that a single miRNA can affect multiple targets via distinct mechanisms^36^. It had been reported the therapeutic usefulness of inhibition of Wnt/β-catenin signaling in cancers^27,37,38^. Moreover, studies had revealed that *miR-26a* inhibited cell proliferation, metastasis, EMT, β-catenin and enhanced apoptosis, E-cadherin via mediating WNT5A in cancers^6,27,39^. The current study demonstrated the function of *miR-26a-5p* in regulating UTUC might mediate through inhibition of WNT5A/β-catenin signaling. Recently, various miRNA replacement therapies are currently in clinical trial demonstrates the great potential of this approach to treat cancer^40^. Therefore, *miR-26a-5p* may be a novel therapeutic small molecule against UTUC.

**Figure 7:**
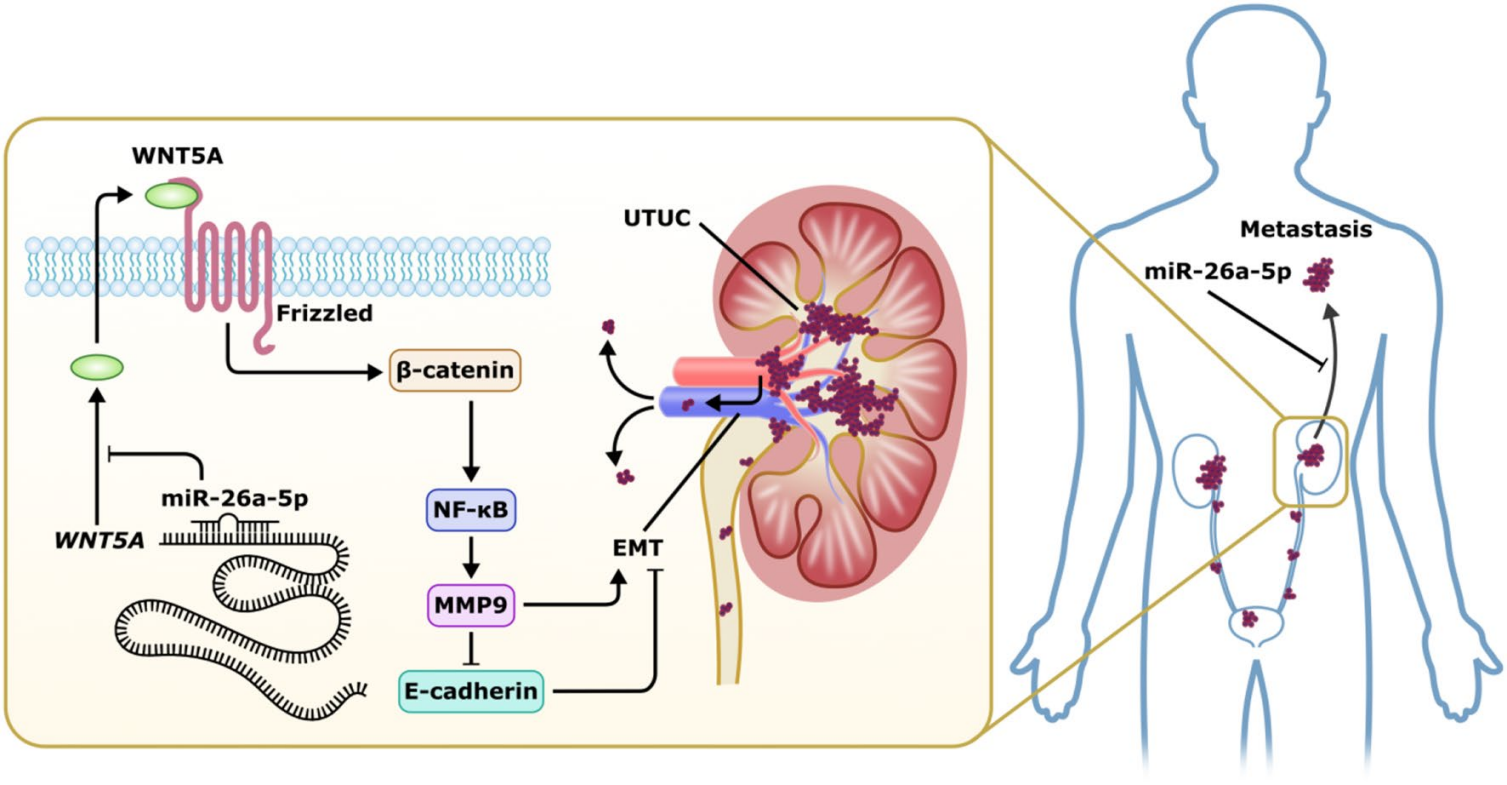
Proposed mechanism for the effects of *miR-26a-5p* on WNT5A/β-catenin signaling in tumorigenesis of upper tract urothelial carcinoma (UTUC). Our findings revealed that *miR-26a-5p* gene delivery may induce simultaneous suppression of WNT5A and β-catenin expression and their downstream NF-κB and MMP-9 proteins. The suppression of WNT5A/β-catenin signaling axis by *miR-26a-5p* restoration could reverse the processes of epithelial-to-mesenchymal transition (EMT) of UTUC cells, including E-cadherin disruption, thereby suppressing their metastasis activity.

In conclusion, the present study provides the evidence to support the anticancer properties of *miR-26a-5p* and act as tumor suppressors in UTUC cells. The major findings of the present study are summarized as a diagrammatic depiction (Fig. 7): *miR-26a-5p* down-regulation in UTUC, which may block WNT5A/β-catenin signaling and inhibit EMT process. Thus, replacement therapy with *miR-26a-5p* represents a promising novel therapeutic strategy against UTUC.

## Materials and methods

### Clinical specimens

All human UTUC tumor tissues (n = 22) and adjacent normal tissues (n = 14) were obtained in an UTUC cohort study in Kaohsiung Chang Gung Memorial Hospital, which was approved by the Institutional Review Board of Chang Gung Memorial Hospital (IRB no. 201801703B1 and 101-4722B) and complied with the Helsinki Declaration. The written informed consent was obtained from all subjects or if subjects are under 18, from a parent/ legal guardian.

### Human UTUC tissue microarray (TMA)

The human renal pelvis transitional cell carcinoma TMA containing 64 cases was purchased from US Biomax, Inc. (Kit no.KD642, Rockville, MD). The TMA slide was subjected to in situ hybridization (ISH) staining of *miR-26a-5p*, and quantified by pathologist according to the following rules. The labeling intensity was given a score from 1 to 4, corresponding to yellow, yellow–brown, brown and dark staining, respectively. The proportion of tumor cells with detectable cytoplasmic immunoreactivity for *miR-26a-5p* were also recorded using a 4-tier score, for 1 corresponding to < 30% tumor cells with positive staining, 2 for 30–60%, 3 for 60–80%, and 4 for > 80%. An expression index was defined as the product of these two scores mentioned above. Obviously, the index could range from 1 to 16, with 16 corresponding to > 80% tumor cells displaying dark (4) staining.

### miR-26a-5p in situ hybridization

TMA slide was examined using in situ hybridization (ISH) staining. Tissue sections were stained with digoxigenin-labeled probe (sequence 5ʹ-AGC CTA TCC TGG ATT ACT TGAA-3ʹ) synthesized by BioTnA and complimentary to *miR-26a-5p* The expression level was measured by using a Biospot ISH detection kit (TASH01D, BioTnA, Kaohsiung, Taiwan). The signals were developed by DAB chromogen and documented under microscope as described^41^.

### RNA isolation, reverse transcription and quantitative PCR (qPCR)

Total RNA was extracted by using Trizol^®^ Reagent (Invitrogen, USA) according to the instruction manual as previously described^3^. To prepare a cDNA pool from each RNA sample, total RNA (10 ng) was reverse-transcribed using TaqMan MicroRNA reverse transcription kit (ABI, Cat. 4366596) according to the manufacturer’s instructions as previously described^3^. The primers are listed on Supplementary Table 1.

### Cell culture and miR-26a-5p treatment

A UTUC cell line, clone BFTC-909, was cultured as described previously^3^. To observe the biomodulatory effect of *miR-26a-5p* on UTUC cell behaviors, the cells were stably transfected with *miR-26a-5p* mimics following the manufacturer’s instructions and the Guidelines for miRNA mimic and miRNA inhibitor experiments (QIAGEN, Hilden, Germany).

### Cell proliferation assay

Cell proliferation assay was performed by using WST-1 assay kit (TaKaRa Cat. # MK400). All procedures were followed as previously described^3^.

### Trans-well cell migration assay

Cell migration assay was performed using Trans-well chambers (Millicell, PIEP12R48). All procedures were followed as previously described^3^.

### Western blot analysis

Cellular protein lysates were prepared by homogenization with 1× RIPA lysis buffer (Cell Signaling Technology, Billerica, MA). Protein concentrations were measured using a protein assay dye (Bio-Rad Laboratories, Hercules, CA). SDS-PAGE and immunoblotting analysis were performed as described previously^3^. The detecting antibodies were raised against E-cadherin (ab76055, abcam), vimentin (2707-1 epitomics), α-SMA (ab5694, abcam), fibronectin (ab2413, abcam), WNT5A (MA5-15502, Invitrogen), β-catenin (13-8400, Invitrogen), NF-κB (ab32536, abcam), MMP-9 (13667, CST), and GAPDH (GTX627408, GeneTex).

### Immunofluorescent staining

BFTC909 cells were cultured in six-well glass slide chambers for 24 h and further transfected with *miR-26a-5p* mimics for 48 h. The cells were then fixed with 4% paraformaldehyde, permeabilized with 0.25% Triton X-100, and blocked with 3% BSA for 30 min at room temperature. The fixed cells were then incubated with the primary antibodies against E-cadherin, vimentin, fibronectin, α-SMA, WNT5A, β-catenin, NF-κB, and MMP-9 at 4 °C overnight followed by visualization with Alexa Fluor 488- (green) or Alexa Fluor 595 (red)-conjugated secondary antibodies at room temperature for 1 h. Nuclei were counterstained with DAPI. The stained cells were mounted with a fluorescent mounting medium (Dako Cytomation) and observed under fluorescence microscopy (Olympus). The exposure gains and rates were consistent between samples. Fluorescent intensities were quantified on independent color channels by using Image J software (NIH, USA)^3^.

### Statistical analysis

All values in the figures were expressed as mean ± standard error of the mean and analyzed by unpaired t test. *P* value < 0.05 was considered to be statistically significant.

## Supplementary Information

Supplementary Figures.

Supplementary Table 1.
