# Supplementary material for: MiR-26a-5p as a useful therapeutic target for upper tract urothelial carcinoma by regulating WNT5A/β-catenin signaling
Source: Sci Rep. 2022 Apr 28;12:6955. doi: 10.1038/s41598-022-08091-6 (PMC9050734; doi:10.1038/s41598-022-08091-6)
Supplement: Supplementary file 1 — Supplementary Figures. [file 41598_2022_8091_MOESM1_ESM.docx]

**Supplementary Figures**

**Figure S1.** ***miR-26a-5p* overexpression inhibited expression of epithelial-to-mesenchymal transition markers in UTUC cells.** BFTC-909 UTUC cells were transfected with *miR-26a-5p* mimics for 48 hrs and subjected to mRNA and protein measurements. Expression levels of epithelial marker E-cadherin and mesenchymal markers including vimentin, fibronectin, and α-SMA were assayed with western blot, respectively. GAPDH was used as loading internal control. The raw blots images of full length membranes/gels and the red frames were the image present of figure 4B. The list as followed as control (Con.), Scramble (Scr.) and miR-26a-5p (26a).

**
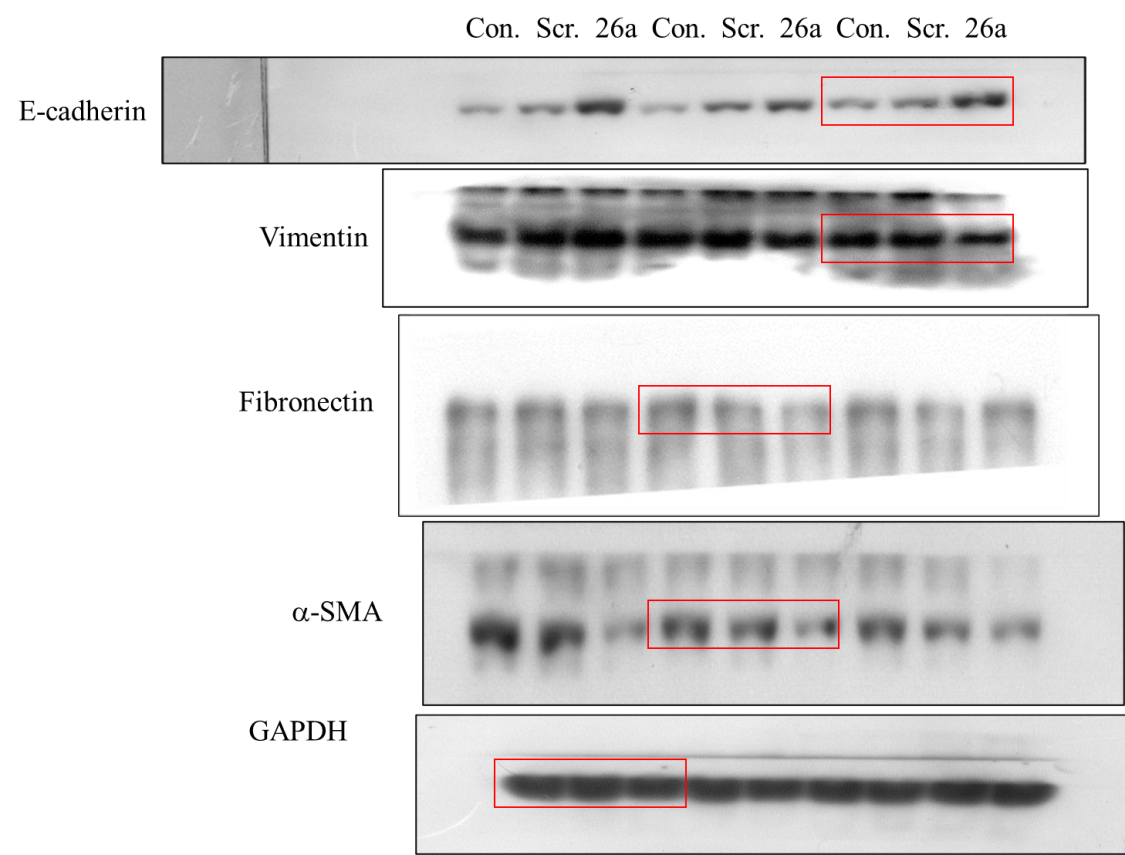
**

**Figure S2. *miR-26a-5p* overexpression modulated expression of Wnt5A/β-catenin signaling mediators and downstream molecules in UTUC cells.** BFTC-909 cells were transfected with *miR-26a-5p* mimics for 48 hrs and subjected to detection protein expression by western blot, respectively. GAPDH was used as loading internal control. The raw blots images of full length membranes/gels and the red frames were the image present of figure 6B. The list as followed as control (Con.), Scramble (Scr.) and miR-26a-5p (26a).

**
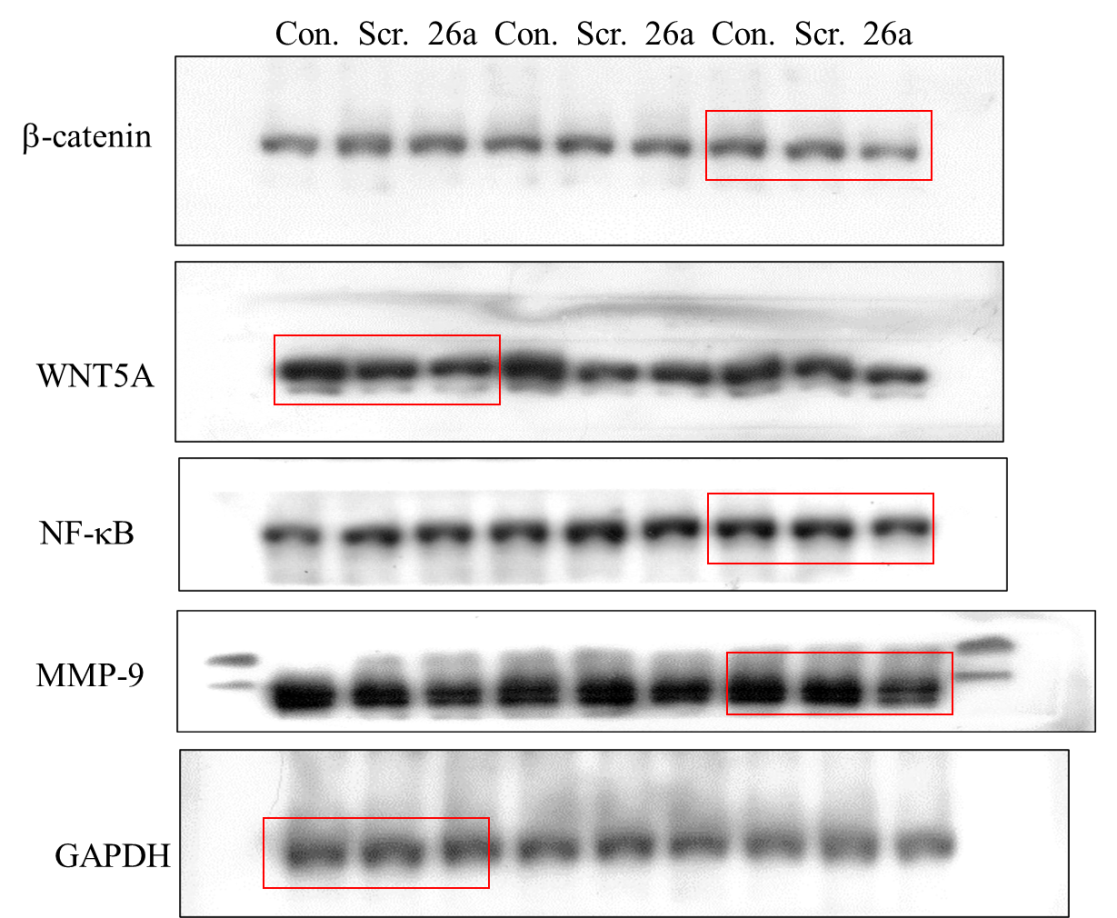
**
