# Supplementary material for: MiR-26a-5p as a useful therapeutic target for upper tract urothelial carcinoma by regulating WNT5A/β-catenin signaling
Source: Sci Rep. 2022 Apr 28;12:6955. doi: 10.1038/s41598-022-08091-6 (PMC9050734; doi:10.1038/s41598-022-08091-6)
Supplement: Supplementary file 2 — Supplementary Table 1. [file 41598_2022_8091_MOESM2_ESM.docx]

Supplementary Table 1. The primer sequences used for qPCR were as follows:

| **Gene** | **Primer** |
| --- | --- |
| **E-cadherin** | Forward primer: TCATGAGTGTCCCCCGGTAT |
|  | Reverse primer: CGGAACCGCTTCCTTCATAG |
| vimentin | Forward primer: GGACCAGCTAACCAACGACAA |
|  | Reverse primer: TTTTCGGCTTCCTCTCTCTGAA |
| **fibronectin** | Forward primer: TCCTGTTGGCACTGATGAAGAA |
|  | Reverse primer: ACCTCTTCCCGAACCTTATGC |
| α-SMA | Forward primer: CGCCCGCCCCACTT |
|  | Reverse primer: GGGTGGTGTTCAGGGAAGCT |
| MMP-9 | Forward primer: CGCTGGGCTTAGATCATTCC |
|  | Reverse primer: GAGGCCGTGGCTCAGGTT |
| NF-κB | Forward primer: AATGGGCTACACCGAAGCAA |
|  | Reverse primer: CCGCTGTCGCAGACACTGT |
| WNT5A | Forward primer: GCTCGCATCCTCATGAACCT |
|  | Reverse primer: ACCCACCTTGCGGAAGTCT |
| **β-catenin** | Forward primer: CATCCCACTGGCCTCTGATAA |
|  | Reverse primer: TGTGCACGAACAAGCAACTG |
| **GAPDH** | Forward primer: CCCACTCCTCCACCTTTGAC |
|  | Reverse primer: TTACTCCTTGGAGGCCATGTG |
